# Etiology of Gastroschisis

**Published:** 2012-10-01

**Authors:** V Raveenthiran

**Affiliations:** Department of Pediatric Surgery, SRM Medical College and Hospital SRM University, Chennai, India.

 (Athena stands for abbreviation of Abstracting and Thoughtful Evaluation of Neonatal Articles; but it is also personified by the contributor. Like Athena of Greek mythology, she distills wisdom from published literature)

If scientific progress depends upon research experiments, experiments themselves depend upon hypotheses. In material science such as Theoretical Physics, even Nobel Prizes have been awarded for hypothetical works [1]. Unfortunately, the healing science is reeling in the rungs of 'Evidence-Based Medicine'. Although a specialized journal 'Medical Hypotheses' tries to bridge this gap, medical science generally frowns upon theorization without observed numerical data. Athena, who is greatly interested in 'Theoretical Medicine', is pleased to note that etiopathogenic puzzle of gastroschisis has recently been elucidated by several hypothetical papers. 

Traditionally gastroschisis is thought to be caused by vascular accident in early embryonic life. But the nature of accident and its cause remain elusive. Recently, Mark Lubinsky [2] has proposed a brilliant hypothesis that the vascular accident could be fetal thrombophilia secondary to maternal estrogen-surge. Estrogen induced thrombosis, estrogen surge during early pregnancy and transplacental influx of maternal estrogen are well known facts in Medicine. Linking these points, Lubinsky has framed a convincing hypothesis. According to him, high maternal estrogen during early pregnancy causes thrombosis of fetal vessels; palmitic acid, which is a byproduct of thrombosis, affects cell signaling and prevents fusion of body wall folds. Thrombosis of fetal vessels accounts for frequent association of bowel atresia with gastroschisis. Estrogenic surge as the cause of thrombophilia explains early onset of gastroschisis and its high incidence in young mothers. It also explains as to why the malformation is relatively uncommon in obese mothers. Racial difference in thrombogenic susceptibility explicates as to why gastroschisis is more common in the Whites despite comparable estrogen surge in the Blacks. Lubinsky elaborates that maternal intake of alcohol and smoking increases serum estrogen levels thereby makes the fetus vulnerable for gastroschisis. Peculiar to gastroschisis is the palmitic acid rich vacuoles in amniotic fluid. Large amount of palmitic acid produced in consequence of fetal thrombosis is phagocytized by amniocytes. These lipid laden cells in amniotic fluid of gastroschisis are otherwise inexplicable in the absence of Lubinsky's hypothesis.

If Lubinsky's hypothesis is extended to exogenous estrogens, it elegantly explains the recent increase in the incidence of gastroschisis in many American and Australian states. For example the incidence of this malformation shows an annual increase of 10% in Washington State [3] and it has doubled in New Zealand between 1996 and 2004 [4]. This rise is attributed to environmental pollutants such as agrochemicals and polycyclic aromatic hydrocarbons (PCAH). Agrochemicals such as endosulfan, DDT, atrazine and benzopyrene exert estrogenic effect by their structural resemblance to endogenous estrogens. Two case-control studies have in fact added value to Lubinsky's hypothesis. Waller et al [5] compared 805 cases of gastroschisis with 3616 controls. Mothers living within 25 km of atrazine polluted sites more frequently suffer fetal gastroschisis than those who live 50 km away. Conception between April and July carried a higher risk of the malformation because of busy agricultural activities during this period and hence increased maternal exposure to atrazine in the first trimester. Athena is curious to note that this observation may explain the cluster occurrence of gastroschisis which is acknowledged by many pediatric surgeons. Lupo et al [6] studied the effect of occupational exposure of mothers to polycyclic aromatic hydrocarbons (PCAH). On comparing 299 cases of gastroschisis and 2993 controls, they noted that mothers exposed to PCAH have high risk of malformed babies. Interestingly, mothers older than 20 years are significantly more affected than those below 20 years of age. This paradox indicates that PCAH may be a weak estrogen.

Even in the absence of estrogen induced thrombosis, interference with cell signaling can still happen if the implicated end product (palmitic acid) accumulates from other sources such as diet. Weiss et al [7] tested whether maternal nutrition has a role in pathogenesis of gastroschisis by studying 13 affected mothers and 9 controls. They found that higher maternal intake of omega-6 polyunsaturated fatty acid (PUFA) is associated with increased risk of gastroschisis. Interestingly, omega-6 PUFA is one of the precursors of palmitic acid through cyclo-oxygenase pathway. Omega-3 and omega-6 PUFA should be balanced in healthy diet. But in 1960s, it was thought that replacing saturated fatty acid with omega-6 PUFA may reduce cardiovascular disease. Consequently, consumption of omega-6 PUFA containing oils such as Soybean oil, sunflower oil and corn oil has increased 1000 folds in USA. It is to be verified whether this dietary change is responsible for the recent rise in the incidence of gastroschisis. 

Athena wonders what to follow as there is conflicting advice from cardiologists and embryologist regarding omega-6 PUFA. Another paper by Paranjothy et al [8] may provide the solution. In a case-control study they found that higher maternal intake of fruits and vegetables and prolonged supplementation of folic acid in first trimester significantly reduces the risk of gastroschisis independent of other risk factors such as smoking. Interestingly, folic acid is known to modulate estrogen receptor response. Thus Lubinsky's hypothesis can be extrapolated to include maternal nutritional deficiency theory of gastroschisis. 

Based on cluster occurrence of gastroschisis, Werler [9] has proposed another interesting hypothesis that it may be due to Ebstein Barr virus (EBV). According to the author, epidemiological details of the malformation parallels that of EBV. EBV is more common in childhood and adolescence. This correlates with high incidence of gastroschisis in young primigravida below 20 years. Werler argues that the association of cigarette smoking and gastroschisis is in fact due to reactivation of EBV by maternal smoking. Seasonal clustering of the malformation correlates well with the epidemics of EBV. More importantly, trivial symptoms of EBV such as sore throat and low grade fever are usually ignored by mothers and the viral malaise is mistaken for fatigue of pregnancy. Although Athena considers Werler's hypothesis weaker than that of Lubinsky, she recommends experiments to explore the role of EBV. 

Hypotheses are not only exciting in unraveling etiological mystery, but they may also aid in clinical care. Matting of bowel loops is a serious problem in the management of gastroschisis. Matted bowel not only poses difficulty in surgical reduction, but also causes malabsorption in the survivors. Pathogenesis of matting is often attributed to irritant effect of amniotic fluid. Athena used to imagine that bowel matting can be avoided if only the irritant nature of amniotic fluid is nullified. Hakguder et al [10] hypothesized that inflammatory reaction can be minimized by diluting the irritants. They further proposed induction of fetal diuresis to achieve dilution of amniotic fluid. This hypothesis was tested in a rat model. Gastroschisis was surgically created in rat fetuses and pregnancy was continued with intra-amniotic injections of furosemide. Serosal thickening and bowel matting was significantly lesser in diuretic treatment group than in untreated animals. Clinical translatability of this interesting hypothetical experiment is yet to be explored. 

Horrobin - the founding editor of Medical Hypotheses - remarked, “The physical and chemical sciences long ago recognized that observations are not superior to hypotheses in generating scientific progress, nor are hypotheses superior to observations. Both are necessary. While the ideal research worker may be one who is equally able to generate hypotheses and to test them experimentally, these sciences have also recognized that such paragons are very rare indeed. Most scientists are much better at either one or the other activity“ [1]. Athena could not agree more. She wishes the Journal Neonatal Surgery to devote few pages in each issue encouraging hypothetical proposals. After all generating a logically sound hypothesis is the first legitimate step of scientific advancement.

## Footnotes

**Source of Support:** Nil

**Conflict of Interest:** The author is a Joint Editor of the journal. But he did not take part in the evaluation or decision making of this manuscript. The manuscript has been independently handled by two other editors.

## References

[1] Horrobin D F (2004). Ideas in biomedical science: reasons for the foundation of Medical Hypotheses. Med Hypotheses.

[2] Lubinsky M (2012). Hypothesis: Estrogen related thrombosis explains the pathogenesis and epidemiology of gastroschisis. Am J Med Genet A.

[3] Chabra S, Gleason C A, Seidel K, Williams M A (2011). Rising prevalence of gastroschisis in Washington State. J Toxicol Environ Health A..

[4] Srivastava V, Mandhan P, Pringle K, Morreau P, Beasley S, Samarakkody U (2009). Rising incidence of gastroschisis and exomphalos in New Zealand. J Pediatr Surg.

[5] Waller SA, Paul K, Peterson SE, Hitti JE (2010). Agricultural-related chemical exposures, season of conception, and risk of gastroschisis in Washington State. Am J Obstet Gynecol.

[6] Lupo P J, Langlois P H, Reefhuis J, Lawson C C, Symanski E, Desrosiers T A (2012). Maternal occupational exposure to polycyclic aromatic hydrocarbons: effects on gastroschisis among offspring in the national birth defects prevention study. Environ Health Perspect.

[7] Weiss LA, Chambers CD, Gonzalez V, Hagey LR, Jones KL (2012). The omega-6 fatty acid linoleic acid is associated with risk of gastroschisis: a novel dietary risk factor. Am J Med Genet A.

[8] Paranjothy S, Broughton H, Evans A, Huddart S, Drayton M, Jefferson R (2012). The role of maternal nutrition in the aetiology of gastroschisis: an incident case-control study. Int J Epidemiol.

[9] Werler M W (2010). Hypothesis: Could Epstein Barr virus play a role in the development of gastroschisis?. Birth Defects Res A Clin Mol Teratol.

[10] Hakguder G, Olguner M, Gurel D, Akgur F M, Flake A W (2011). Induction of fetal diuresis with intraamniotic furosemide injection reduces intestinal damage in a rat model of gastroschisis. Eur J Pediatr Surg.

